# Genetic and Informatic Analyses Implicate *Kif12* as a Candidate Gene within the *Mpkd2* Locus That Modulates Renal Cystic Disease Severity in the *Cys1^cpk^* Mouse

**DOI:** 10.1371/journal.pone.0135678

**Published:** 2015-08-21

**Authors:** Michal Mrug, Juling Zhou, Chaozhe Yang, Bruce J. Aronow, Xiangqin Cui, Trenton R. Schoeb, Gene P. Siegal, Bradley K Yoder, Lisa M. Guay-Woodford

**Affiliations:** 1 Department of Medicine, The University of Alabama at Birmingham, Birmingham, AL 35294, United States of America; 2 Department of Genetics, The University of Alabama at Birmingham, Birmingham, AL 35294, United States of America; 3 Department of Biostatistics, The University of Alabama at Birmingham, Birmingham, AL 35294, United States of America; 4 Department of Pathology, The University of Alabama at Birmingham, Birmingham, AL 35294, United States of America; 5 Department of Cell, Developmental and Integrative Biology, The University of Alabama at Birmingham, Birmingham, AL 35294, United States of America; 6 Division of Biomedical Informatics, Cincinnati Children's Hospital Medical Center, Cincinnati, OH 35229, United States of America; 7 Department of Veterans Affairs Medical Center, Birmingham, AL 35233, United States of America; 8 Center for Translational Science, Children's National Health System, Washington, DC 20010, United States of America; University of Iowa, UNITED STATES

## Abstract

We have previously mapped the interval on Chromosome 4 for a major polycystic kidney disease modifier (*Mpkd*) of the B6(Cg)-*Cys1* ^*cpk*^/J mouse model of recessive polycystic kidney disease (PKD). Informatic analyses predicted that this interval contains at least three individual renal cystic disease severity-modulating loci (*Mpkd1-3*). In the current study, we provide further validation of these predicted effects using a congenic mouse line carrying the entire CAST/EiJ (CAST)-derived *Mpkd1-3* interval on the C57BL/6J background. We have also generated a derivative congenic line with a refined CAST-derived *Mpkd1-2* interval and demonstrated its dominantly-acting disease-modulating effects (e.g., 4.2-fold increase in total cyst area; p<0.001). The relative strength of these effects allowed the use of recombinants from these crosses to fine map the *Mpkd2* effects to a <14 Mbp interval that contains 92 RefSeq sequences. One of them corresponds to the previously described positional *Mpkd2* candidate gene, *Kif12*. Among the positional *Mpkd2* candidates, only expression of *Kif12* correlates strongly with the expression pattern of *Cys1* across multiple anatomical nephron structures and developmental time points. Also, we demonstrate that *Kif12* encodes a primary cilium-associated protein. Together, these data provide genetic and informatic validation of the predicted renal cystic disease-modulating effects of *Mpkd1-3* loci and implicate *Kif12* as the candidate locus for *Mpkd2*.

## Introduction

The polycystic kidney diseases (PKD) are a major cause of end-stage renal disease [1]. Autosomal dominant PKD (ADPKD; MIM 173900) is caused by mutations in the *PKD1* or *PKD2* genes [2–5] and autosomal recessive PKD (ARPKD; MIM 263200) results from mutations in the *PKHD1* gene [6, 7].

While ADPKD and ARPKD are considered to be classical Mendelian traits, the disease phenotypes in both forms of PKD are complex with respect to the severity of renal cystic disease and extrarenal manifestations. Such phenotypic variability is typical even among family members that share identical PKD mutations, suggesting modulating effects of other genetic (i.e., co-inherited modifier genes), epigenetic, or environmental factors (summarized by Mrug *et al* [8]). Among these modulators of PKD progression, co-inherited modifier gene effects are the most tractable for experimental investigation. Indeed, previous studies have identified several quantitative trait locus (QTL) intervals that harbor genetic modifiers of PKD progression.

To date, the most significant QTL that modulates the severity of renal cystic and biliary phenotypes has been mapped to mouse Chromosome (Chr) 4 [9–12]. In previous studies, we have performed intensive analyses of this interval and discriminated three individual QTL effects on Chr 4 [8]. These effects were designated as *Mpkd1*, *Mpkd2* and *Mpkd3* (MGI:3603220–3603222). Identification of specific candidate genes underlying the effects of the *Mpkd1-3* loci has been complicated by the extensive span of the Chr 4 QTL complex (~50 cM corresponding to over 100 Mbp of genomic sequence with ~1000 RefSeq genes). Therefore, we prioritized the analyses of these positional candidates based on the reported expression in early postnatal kidneys and liver, differential renal expression in kidneys with slowly vs rapidly progressive cystic kidney disease, and comparative analyses of genomic sequence in selected candidates. These analyses implicated *Kif12* as a strong positional candidate gene for the *Mpkd2* effects [8]. All of these studies were performed in the well-characterized B6(Cg)-*Cys1* ^*cpk*^/J (B6-*Cys1* ^*cpk*^) mouse model that phenotypically mimics ARPKD [8, 13–16]. The *Cys1*-encoded protein cystin is a primary cilium-associated protein [13].

In the current study, we use congenic strain analyses to fine-map the predicted renal cystic disease-modulating effects of the *Mpkd* loci and provide further supportive evidence implicating *Kif12* as the candidate *Mpkd2* locus based upon genetic, informatic, and immunolocalization analyses.

## Results

### The congenic CAST/EiJ-derived interval containing the *Mpkd1-3* loci modulates renal cystic disease severity

A congenic line homozygous for the CAST/EiJ (CAST)-derived proximal-medial segment of Chr 4 on the C57BL/6J (B6) genetic background (B6.CAST.4PM) was developed previously by mating (B6 × CAST)F1 females with B6 males; the male progeny with the desired microsatellite marker profile were backcrossed to B6 females; mice at the N6 generation or later were intercrossed. Homozygous lines were selected for propagation [17].

**Fig 1:**
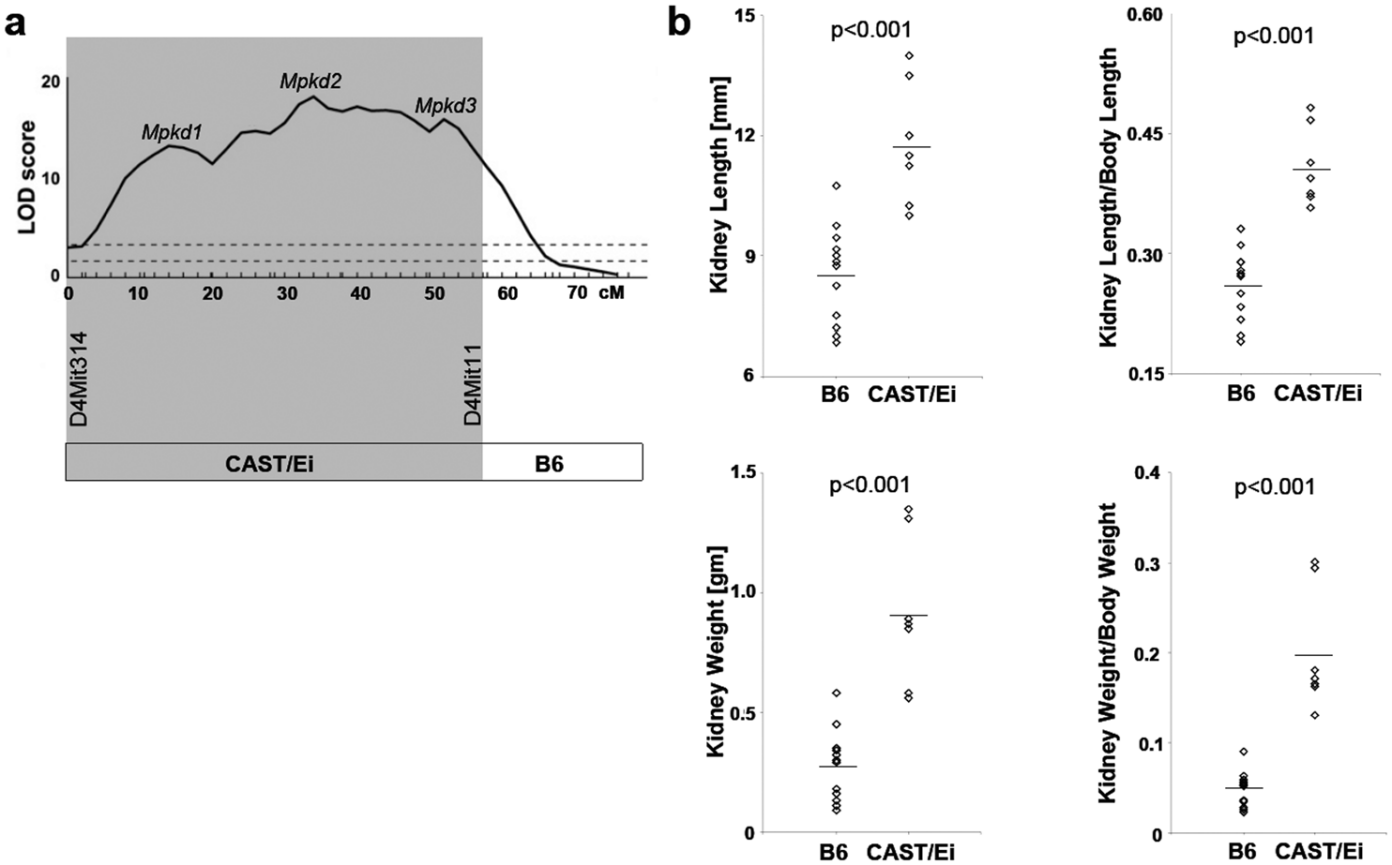
Congenic CAST-derived interval containing the *Mpkd1-3* loci and its effects on renal cystic disease progression. a) The CAST-derived segment of Chr 4 corresponding to *Mpkd1-3* interval is delimited by the distal marker, D4Mit11 (shaded area); the breakpoint between proximal CAST-derived and distal B6-derived segment of Chr 4 occurred between the markers, D4Mit11 (57.4 cM) and D4Mit204 (61.2 cM). b) The predicted cystic disease-modulating effects of the CAST-derived *Mpkd1-3* loci were tested by comparing surrogates of renal cystic phenotypes (kidney length and weight) in *Cys1* ^*cpk/cpk*^ mutants homozygous for the CAST-derived (CAST/Ei; n = 7) vs the B6-derived (B6; n = 12) segment of Chr 4. The genetic background for both groups was B6. Diamonds represent values for individual animals. The line indicates the mean value for each group.

We used a series of microsatellite markers to confirm the CAST origin of the Chr 4 interval in the B6.CAST.4PM strain, to validate the B6 origin of the other Chromosomes, and to fine-map the break point between the proximal CAST and distal B6 intervals on Chr 4, defined by D4Mit11 and D4Mit204 (57.4–61.2 cM). We then interogressed the *Cys1* ^*cpk*^ mutation into this mouse line using a (B6.CAST.4PM x B6-*Cys1* ^*cpk/+*^) backcross and established a new line, B6.CAST.4PM-*Cys1* ^*cpk/+*^. We used the same backcross to generate a control *Cys1* ^*cpk/+*^ line in which the entire length of Chr 4 was derived from the B6 strain (B6.4PM-*Cys1* ^*cpk/+*^). Since the CAST-derived Chr 4 interval in B6.CAST.4PM-*Cys1* ^*cpk/+*^ line spans the three *Mpkd1*, *Mpkd2* and *Mpkd3* loci (Fig 1a), this congenic line allowed us to more precisely evaluate the CAST-derived effects that were identified in our previous studies.

In our initial studies, we characterized the renal cystic disease severity of 10-d old *Cys1* ^*cpk/cpk*^ mice generated from the B6.CAST.4PM-*Cys1* ^*cpk/+*^cross and compared it to the renal phenotypes of *Cys1* ^*cpk/cpk*^ mice generated from the B6.4PM-*Cys1* ^*cpk/+*^cross. These analyses revealed strong disease-modulating effects of CAST-derived *Mpkd1-3* loci, as evidenced by four indices of cystic disease severity (Fig 1b). Specifically, kidney length (KL) increased 1.4-fold; total kidney weight (KW), increased 3.3-fold (both p<0.001 by t-test; n = 12 for B6 and n = 7 for CAST-derived *Mpkd1-2* loci); KL adjusted for body length (crown to rump; BL), the KL/BL ratio, increased 1.6-fold; and KW adjusted for body weight (BW), the KW/BW ratio, increased by 4.2-fold (both p<0.001). These results are consistent with strong renal cystic disease-modulating effects of the composite *Mpkd1-3* locus. Since our experimental design compared the impact of homozygosity for CAST-derived vs B6-derived *Mpkd1-3* loci, the observed differences reflect the sum of various (e.g., dominant, recessive, and additive) disease phenotype-modifying *Mpkd1-3* effects.

### The CAST-derived *Mpkd1-2* locus has a dominantly-acting renal cystic disease accelerating effect

**Fig 2:**
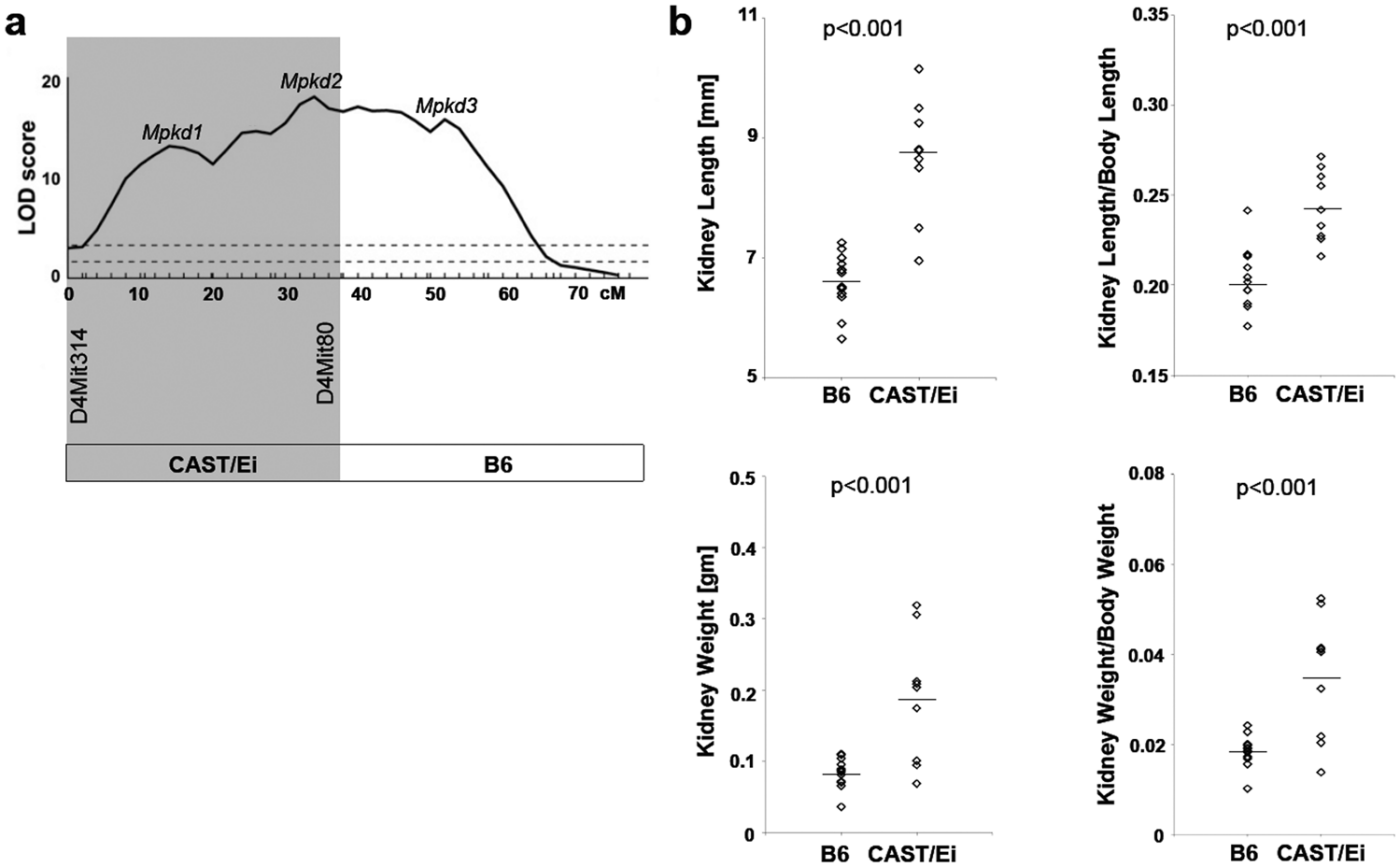
Congenic CAST-derived interval containing the *Mpkd1-2* loci and its effects on renal cystic disease progression. a) The CAST-derived segment of Chr 4 corresponding to *Mpkd1-2* interval is delimited by the marker, D4Mit80 (shaded area). The breakpoint between the proximal CAST/E-derived and distal B6-derived segment of Chr 4 occurred between markers D4Mit80 (37.7 cM) and D4Mit175 (45.7 cM). b) The predicted cystic disease-modulating effects of the CAST-derived *Mpkd1-2* loci was tested by comparing the renal cystic phenotypes in *Cys1* ^*cpk/cpk*^ mutants that were heterozygotes for the CAST-derived (CAST/Ei; n = 9) *Mpkd1-2* interval to *Cys1* ^*cpk/cpk*^ mutants from the same cross that were homozygous B6 for the *Mpkd1-2* interval (n = 14). The genetic background of both groups was B6. Diamonds represent values for individual animals. The line indicates the mean value for each group.

Our previous studies suggested strong interactions among the individual *Mpkd1-3* loci [8]. As the first step towards determining whether these loci may also act independently, we generated a new *Cys1* ^*cpk/+*^ congenic line, B6.CAST.4PM.P1, in which the CAST-derived Chr 4 interval included only the proximal segment of Ch 4 that contains *Mpkd1* and *Mpkd2*, but not *Mpkd3* (Fig 2a). The breakpoint between the proximal CAST and distal B6 intervals in this new line is defined by D4Mit80 and D4Mit175 (37.7 cM and 45.7 cM).

To evaluate a subset of dominant renal cystic disease-promoting effects associated with CAST-derived *Mpkd1-2* loci, we generated *Cys1* ^*cpk/cpk*^ mice heterozygous for the CAST-derived *Mpkd1-2* loci and compared their renal cystic disease severity with *Cys1* ^*cpk/cpk*^ mice homozygous for B6-derived *Mpkd1-2* loci. These two groups were generated in the same B6.CAST.4PM.P1 x B6-*Cys1* ^*cpk/+*^ backcross. Phenotypic analysis at post-natal day 10 revealed strong disease-accelerating effects of CAST-derived *Mpkd1-2* loci, as evidenced by our indices of cystic disease severity (Fig 2a). Specifically, KL increased by 1.3-fold; KW increased by 2.3-fold (both p<0.001 by t-test; n = 14 for B6- and n = 9- for CAST-derived *Mpkd1-2* loci); the KL/BL ratio increased by 1.2-fold; and the KW/BW ratio increased 1.9-fold (both p<0.001). These results are consistent with a dominantly-acting, cystic disease-modulating effect of the CAST-derived *Mpkd1-2* loci.

Similar to the weight- and length-derived phenotypes (i.e., KW and KL), renal cystic indices support the dominantly-acting cystic disease-modulating effects of the CAST-derived *Mpkd1-2* locus. The total cyst area increased 4.2-fold, with the cyst area of the medulla increasing 3.9-fold and the cyst area of the cortex increasing 5.6-fold (all p<0.001). However, the CAST-derived *Mpkd1-2* locus had no effect on total cyst number or number of cysts in either the medulla or the cortex (Table 1). These data suggest that the *Mpkd1-2* loci do not promote renal cystogenesis *per se*, but rather modulate renal cystic disease severity.

**Table 1:** Renal cystic indices of mice with one CAST/Ei-derived and one C57BL/6J-derived *Mpkd1-2* locus (CAST/Ei.B6) vs. two C57BL/6J-derived *Mpkd1-2* loci (B6.B6)

*Mpkd1-2* locus		Total Cyst Number	Total Tissue Area (TA)	Total Cyst Area (CA)	% CA/TA	Medulla Cysts Number	Medulla Area (MA)	Medulla Cyst Area (MCA)	% MCA/MA	Cortex Cysts Number	Cortex Area (CoA)	Cortex Cyst Area (CoCA)	% CoCA/ CoA
B6.B6	average	554	13.4	1.9	14.2	371	6.9	1.6	23.6	184	6.6	0.3	4.3
	SEM	14	0.2	0.0	0.2	9	0.1	0.0	0.3	6	0.1	0.0	0.1
CAST/Ei.B6	average	532	19.7	7.9	37.1	335	11.8	6.4	50.2	197	7.8	1.6	17.9
	SEM	11	0.7	0.5	1.4	8	0.5	0.4	1.5	6	0.2	0.1	1.2
	*p-value*	*0*.*750*	*0*.*002*	*<0*.*001*	*<0*.*001*	*0*.*424*	*0*.*001*	*<0*.*001*	*<0*.*001*	*0*.*659*	*0*.*057*	*<0*.*001*	*<0*.*001*

all reported areas are in mm^2^; SEM = standard error of the mean; *p-value* = significance of differences between the B6.B6 and CAST/Ei.B6 averages

### Fine mapping of the *Mpkd2* effects

**Fig 3:**
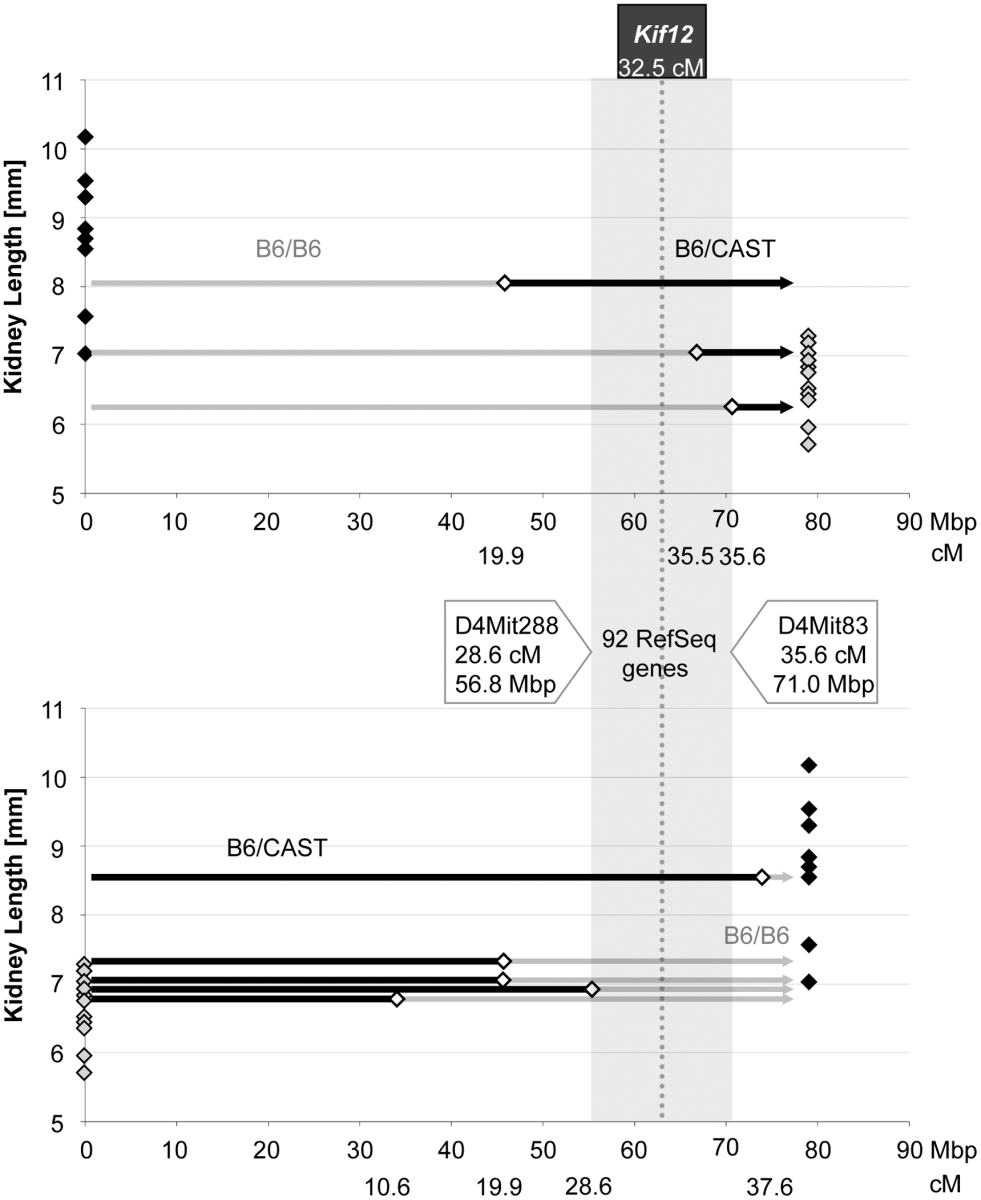
Fine-mapping of renal cystic disease progression-modulating *Mpkd2* effects. We adapted a deletion mapping approach to fine map the dominant cystic disease-modulating effects of the CAST-derived *Mpkd2* locus using genetic recombinants for the *Mpkd1-2* interval that were generated from the B6.CAST.4PM.P1-*Cys1* ^*cpk/+*^ x B6-*Cys1* ^*cpk/+*^ cross. Specifically, we refined the boundaries of the *Mpkd2* interval with CAST-containing *Mpkd1-2* recombinants whose phenotypes (i.e., kidney length) were less than ~2 SD of the phenotype distribution in the CAST *Mpkd1-2* interval homozygotes (black-filled diamonds). The *Mpkd1-2* interval is designated by light shading on upper panel). The refined *Mpkd2* interval (dark shaded area) is delimited by D4Mit288 (28.6 cM, 56.8 Mbp) and D4Mit83 (35.6 cM, 71.0 Mbp). It contains 92 RefSeq sequences and *Kif12* maps to the center of this interval (dashed line). Consistent with the predicted cystic disease-modulating effects of the *Mpkd2* locus, recombinants carrying the refined *Mpkd2* interval of CAST origin had more severe phenotypes (e.g., increased kidney length by 20%; p = 0.004) when compared to recombinants that did not carry the CAST-derived *Mpkd2* interval.

We adapted a deletion mapping approach [18, 19] to fine map the renal cystic disease-modulating effects of the *Mpkd2* locus using 10-d old *Cys1* ^*cpk/cpk*^ *Mpkd1-2* recombinants that were generated from the B6.CAST.4PM.P1 x B6-*Cys1* ^*cpk/+*^ backcross (Fig 3). Using this approach, we narrowed the *Mpkd2* interval to 14 Mbp defined by D4Mit288 and D4Mit83 (Chr 4 position 56,769,379 and 70.962.376). This interval contains 175 gene entries in the NCBI Map Viewer (http://www.ncbi.nlm.nih.gov; NCBI Mus musculus Annotation Release 104), of which 116 are RefSeq sequences and 59 sequences correspond to predicted, but as yet uncharacterized genes. Among the RefSeq sequences, there are 92 protein-encoding genes, 3 microRNAs, and 21 pseudogenes.

We focused our subsequent evaluation of positional *Mpkd2* candidates on the subset of the RefSeq sequences that are expressed in the kidney and liver, the two organs that predominantly express the recessive PKD phenotypes [8]. Based on NCBI Unigene data (http://www.ncbi.nlm.nih.gov), 33 of these RefSeq sequences are expressed in kidney and liver (Table 2), and of these, 19 contain a single nucleotide polymorphism (SNP) variant based on comparison of B6 vs CAST genome sequence data (Wellcome Trust Sanger Institute, Mouse Genome Project Data querying and visualization tool; http://www.sanger.ac.uk). Among these 19 sequences, 11 genes (*Ikbkap*, *Svep1*, *AI314180*, *Fkbp15*, *Hdhd3*, *Kif12*, *Col27a1*, *Akna*, *Whrn*, *Tlr4* and *Cdk5rap2*) contain at least one amino acid change that in the context of disease would be predicted to be deleterious using software-based algorithms [20, 21]. In addition, among the 33 kidney and liver expressed RefSeq sequences mapped to the *Mpkd2* interval, 6 genes (*Ikbkap*, *Svep1*, *Fkbp15*, *Kif12*, *Col27a1 and Tlr4*) contain an in frame insertion or deletion variant.

**Table 2:** Positional candidates for *Mpkd2* effects: a subset expressed in kidney and liver.

Symbol	start	stop	Kidney [TPM]	Liver [TPM]	Neonate [TPM]	Missense SNP variant	Predicted pathogenicity of the AA change	Inframe insertion or deletion variant in CAST (vs B6)
***Ikbkap***	**56763646**	**56815131**	**24**	**9**	**46**	**8**	**+**	**single AA del**
*Ctnnal1*	56823807	56878060	56	9	65	1	-	-
*Epb4*.*1l4b*	53941976	54093158	8	62	14	3	-	-
*Txn1*	57956245	57969283	96	45	84	-	-	-
***Svep1***	**58055668**	**58219468**	**16**	**9**	**74**	**15**	**+**	**single AA ins**
***AI314180***	**58812902**	**58925597**	**120**	**45**	**112**	**3**	**+**	**-**
*Gng10*	59048028	59054771	24	18	65	-	-	-
*Ugcg*	59189203	59222833	32	8	166	-	-	-
*Hsdl2*	59594463	59631566	282	36	46	-	-	-
*E130308A19Rik*	59639199	59767175	8	9	46	-	-	-
*Inip*	59769642	59783855	48	17	55	-	-	-
*Mup4*	59969678	59973537	8	7133	-	1	-	-
*Mup9*	60418046	60421958	24	1849	-	-	-	-
*Mup1*	60510886	60514832	32	4625	-	-	-	-
*Slc31a2*	61947479	61959445	32	9	28	1	-	-
***Fkbp15***	**62300342**	**62360591**	**48**	**71**	**9**	**3**	**+**	**single AA del x1, ins x2**
*Slc31a1*	62021783	62052796	120	162	84	1	-	-
*Cdc26*	62055623	62069657	32	63	56	-	-	-
*Prpf4*	62069817	62088024	32	9	28	-	-	-
***Hdhd3***	**62160088**	**62163234**	**16**	**126**	**9**	**2**	**+**	-
*Alad*	62170204	62181097	137	298	93	-	-	-
*Pole3*	62184832	62186048	24	27	18	-	-	-
*Rgs3*	62220881	62363369	24	36	121	4	-	-
*Ambp*	62804313	62815176	8	2018	-	2	-	-
***Kif12***	**62826671**	**62833165**	**282**	**9**	**280**	**3**	**+**	**five-AA ins**
***Col27a1***	**62876446**	**62996025**	**16**	**27**	**56**	**13**	**+**	**six-AA del**
***Akna***	**63028159**	**63064388**	**48**	**9**	**46**	**11**	**+**	**-**
***Whrn***	**63075944**	**63156985**	**8**	**8**	**27**	**2**	**+**	**-**
*Atp6v1g1*	63205871	63211735	80	63	18	-	-	-
*6330416G13Rik*	63221390	63247389	32	9	9	-	-	-
***Tlr4***	**66488845**	**66503831**	**16**	**18**	**74**	**9**	**+**	**single-AA del**
***Cdk5rap2***	**69884058**	**70071401**	**40**	**9**	**28**	**12**	**+**	-
*Megf9*	70092961	70195962	8	36	224	3	-	-

"TPM"—transcripts per million reported at UniGene; "AA"—amino acid

### Among the *Mpkd2*-associated genes, *Kif12* expression most closely correlates with the structural and developmental expression pattern of *Cys1*

**Fig 4:**
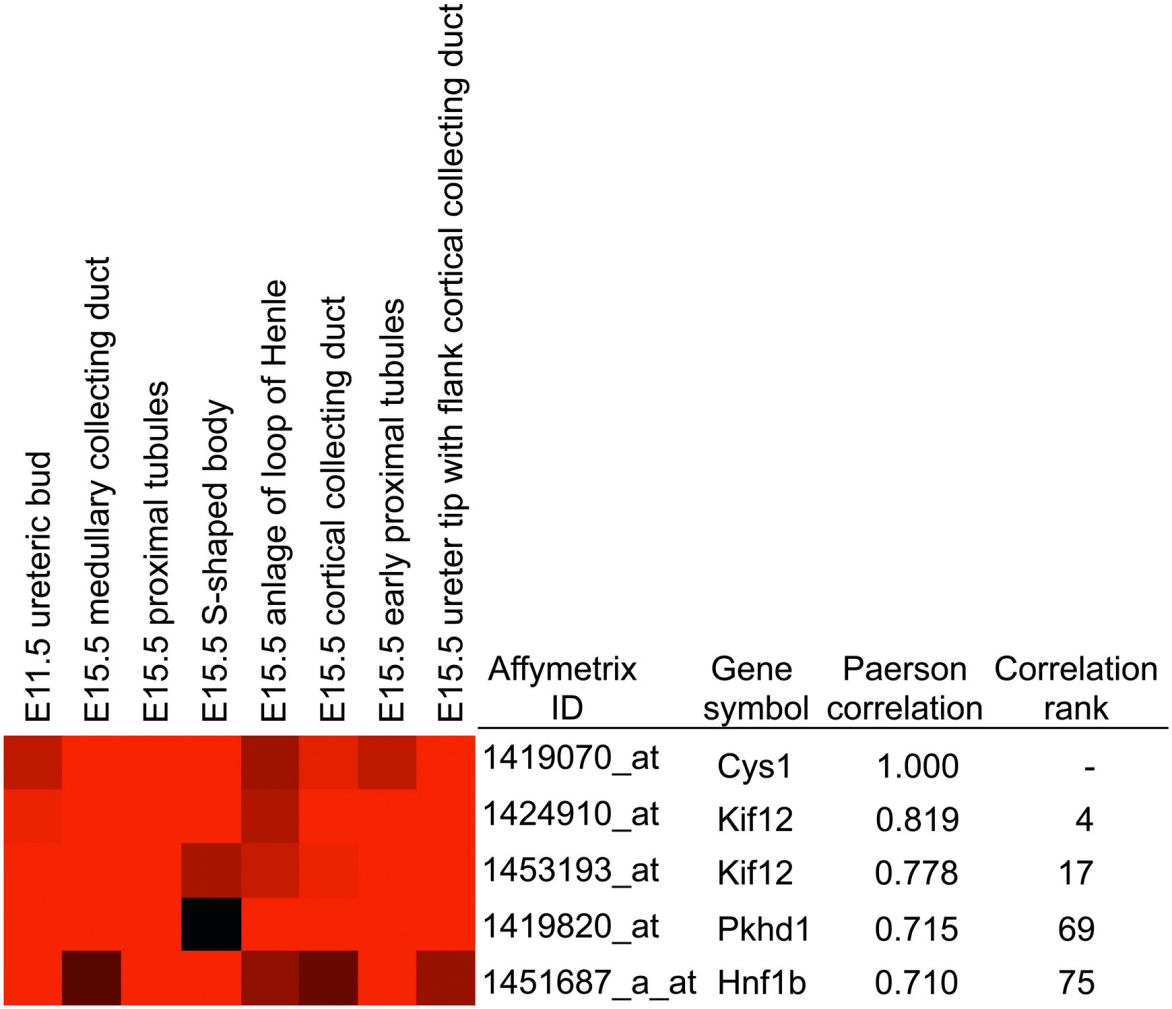
*Kif12* expression pattern mimics expression of *Cys1*. Among ~40,000 transcripts profiled in 18 different renal anatomical structures at different developmental time-points that were deposited into the GUDMAP Database, *Kif12* was the only gene from the *Mpkd2* interval whose expression was highly correlated with that of *Cys1*. Other non-Chr 4 genes with highly correlated expression patterns included *Pkhd1*, the mouse orthologue of the gene mutated in virtually all cases of human ARPKD, and *Hnf1b*, that encodes a direct transcriptional regulator of *Pkhd1*, *Cys1* and *Kif12*. In addition, *Hnf1b* mutations result in a renal cystic phenotype (MODY5 syndrome; MIM 137920). The heatmap depicts a subset of structures and developmental stages highly relevant to renal cystic disease. Red shading corresponds to higher expression values; darker shading to lower values.

To evaluate the relationships between the renal expression patterns of *Cys1* and the genes that map to the *Mpkd2* interval, we compared the transcriptional profiles across 18 different nephron-derived anatomical structures and several development time points using the Genitourinary Molecular Anatomy Project (GUDMAP) Database [http://www.gudmap.org; [22]]. Among the top 100 of the ~40,000 GUDMAP transcript entries that most closely correlate with the *Cys1* expression pattern (S1 Fig), *Kif12* ranked fourth in rank correlation (r = 0.819) and was the only gene that mapped to the *Mpkd2* interval. Based on a comparison that used *Cys1* as the query to which all other transcripts in the GUDMAP Database were compared, the *Cys1* expression pattern was also highly correlated with the expression of several key cystogenic genes (Fig 4), including *Pkhd1* (correlation rank 69), the orthologue of principal human ARPKD gene, and *Hnf1b* (correlation rank 75), which encodes the transcription factor, hepatocyte nuclear factor-1beta (HNF1B) that directly regulates the expression of several cystogenic genes including *Pkhd1* [summarized by Igarashi *et al*. [23]], *Cys1* [24] and *Kif12* [25]. Reciprocal analyses using *Kif12* as the query revealed that *Kif12* expression is correlated most strongly with that of the *Cys1* gene. *Kif12* expression was also highly correlated with the expression of *Pkhd1* (correlation rank 26), and *Hnf1b* (correlation rank 15).

### The *Mpkd2* locus-associated gene *Kif12* encodes a primary cilium-associated protein

**Fig 5:**
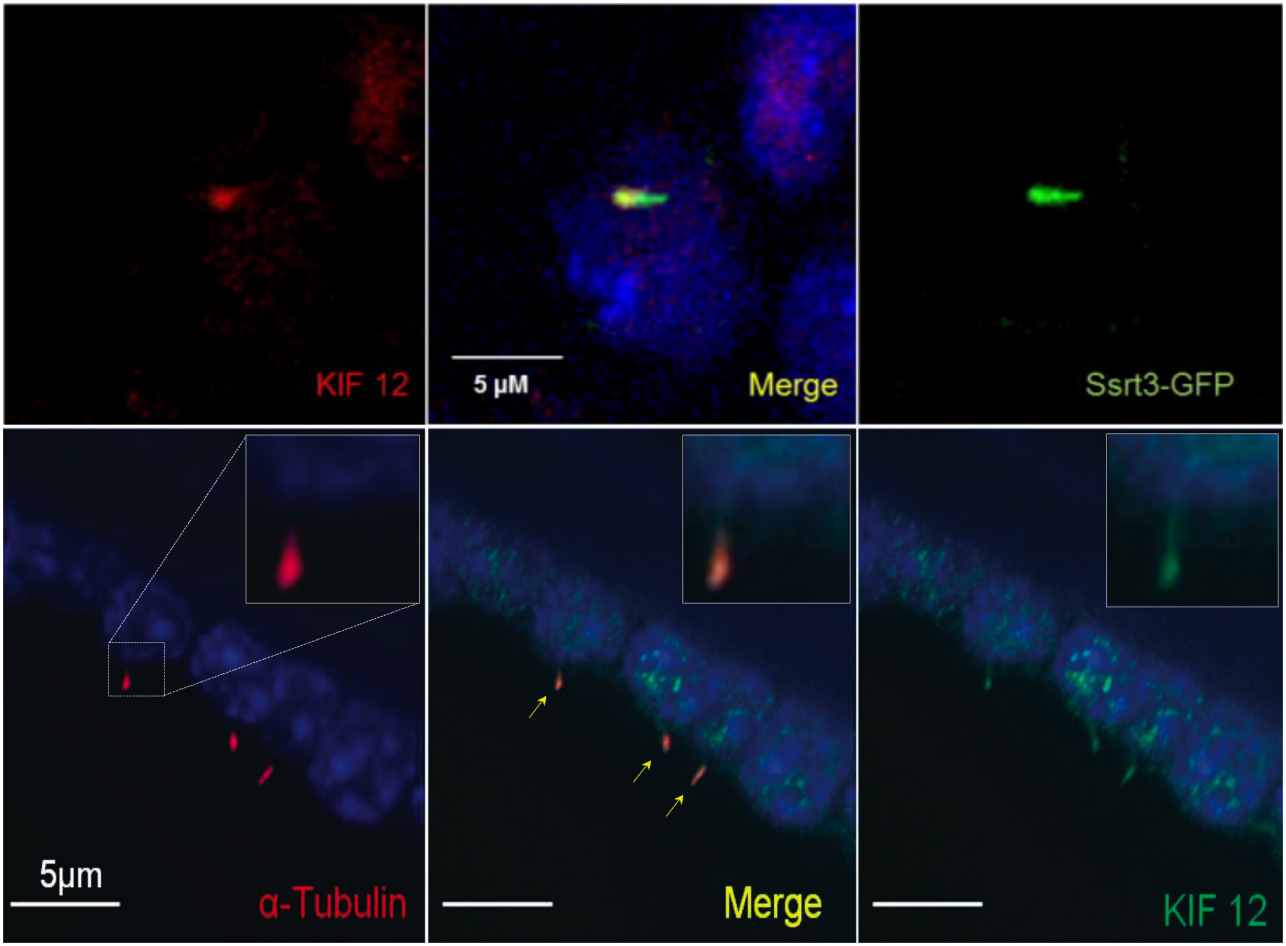
Kinesin 12 localizes to primary apical cilium. **Upper panel** shows representative immunofluorescent micrographs of kinesin 12 (*Kif12*; red) co-localized with a GFP-tagged primary cilia marker, somatostatin receptor 3 (*Sstr3*-GFP; green) in a mIMCD cell line expressing *Sstr3*-GFP. **Lower panel** demonstrates co-localization of kinesin 12 with **α**-tubulin, another commonly used primary cilia marker. The right upper corner insert represents magnification of the left uppermost primary apical cilium (highlighted in the lower **left panel**). Together, these data implicate kinesin 12 as a primary cilia-associated protein.

We have also demonstrated that the *Kif12*-encoded protein, member 12 (kinesin 12), co-localizes with the primary cilia markers somatostatin receptor 3 [26] and α-tubulin in a principal cell line derived from the mouse internal medullary collecting duct (mIMCD; Fig 5). This observation provides the first evidence that an *Mpkd2* locus-associated gene encodes a primary cilium-associated protein. Since the protein product of *Cys1* localizes to primary cilium together with most genes involved in renal cystic disease, localization of the *Kif12* protein product to primary cilia provides further support for this gene as an *Mpkd2* locus candidate.

## Discussion

The strong renal cystic disease-modulating effects observed in *Cys1* ^*cpk/cpk*^ mice carrying the CAST-derived *Mpkd1-3* interval or its *Mpkd1-2* derivative, provide further validation of our initial QTL mapping studies that identified a major effect QTL on Chr 4 [8]. Importantly, the current study demonstrates that the *Mpkd1-3* complex exerts its effects in a fashion that is independent of previously identified non-Chr 4 QTL [8]. In addition, while our previous studies predicted interactions among genes in the Chr 4 QTL complex [8], the current study shows that an isolated *Mpkd1-2* interval, without the *Mpkd3* locus, is sufficient to exert a strong, dominantly-acting effect on renal cystic disease severity. This *Mpkd1-2* QTL complex also modulated the cystic area in the renal cortex and medulla, but did not influence cyst number. Therefore, we propose that the *Mpkd1-2* locus most likely modulates progression of existing renal cystic disease, rather than initiating new cyst formation, which may be regulated by other factors known to impact cyst number, such as the renoprotective enzyme, heme oxygenase-1 [16].

We then went on to determine whether the *Mpkd2* interval alone contains a gene (or multiple genes) with strong cystic disease-modulating effects. We developed a complementary set of strategies to assess whether the *Mpkd2* effect could result from a coding sequence variant within a subset of positional gene candidates that are expressed in kidney and liver, the two organs that predominantly express the recessive PKD phenotypes. This approach identified 11 promising positional *Mpkd2* candidates.

While multiple coding variant changes are present within the 11 genes that map to the this interval (Table 2), only the expression of the previously described candidate, *Kif12* [8], is strongly correlated with the expression of *Cys1* across the multiple anatomical nephron structures and developmental time points catalogued in the GUDMAP Database. While a complete systematic evaluation of each *Mpkd2* positional candidate on protein level is confounded by limited immunoreagents, we have demonstrated that kinesin 12, the protein encoded by *Kif12*, is a primary cilia-associated protein. Together, these data implicate *Kif12* as a candidate genetic modifier within the *Mpkd2* interval.

**Fig 6:**
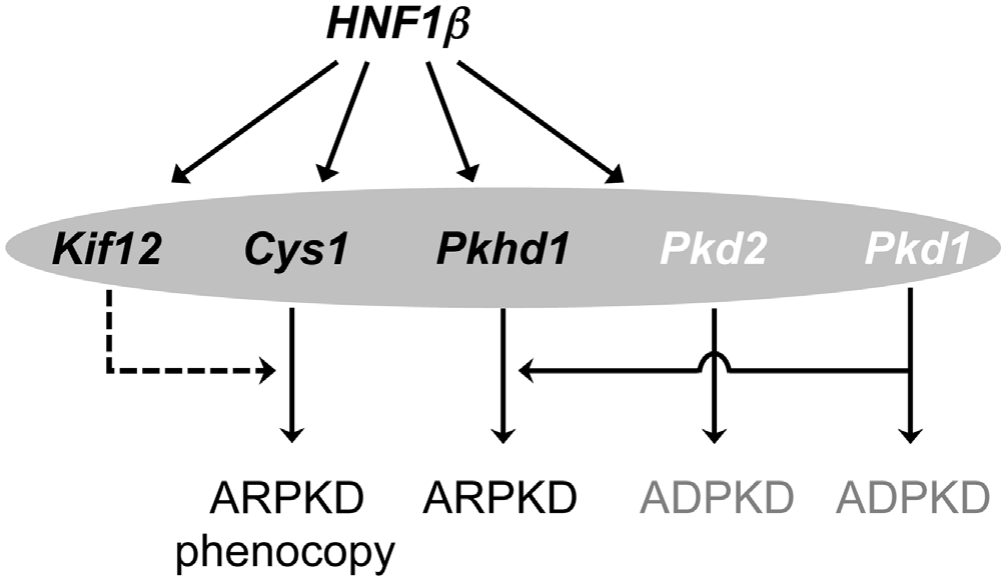
Proposed integration of *Kif12* into recessive PKD genetic pathway. *Hnf1 β* regulates the transcription of several cystogenic genes including *Pkhd1*, the mouse orthologue of the principal ARPKD gene; *Cys1*, the gene mutated in the *cpk* model of ARPKD, and *Kif12*, a candidate modifier of the *Cys1* ^*cpk/cpk*^ phenotype. Therefore, we propose that the four proteins encoded by *Hnf1b*, *Cys1*, *Kif12*, and *Pkhd1* may define a functional complex that modulates the progression of recessive PKD (black font). This hypothesis is supported by the very high correlations among structural and developmental expression patterns of these four genes (Fig 4). In addition, components of the recessive PKD-associated pathway may interact at different levels with functional components of ADPKD-associated pathways (white font). For example, *HNF1β* also regulates the expression of *Pkd2*, an ADPKD gene. Furthermore, genetic interaction studies suggest that abnormal *Pkd1* expression sensitizes renal tubular cells to *Pkhd1* defects [28, 29]. The latter interaction may physically occur within the primary apical cilium (shaded oval), where these proteins all co-localize. The proposed *Kif12* modulating effect on *Cys1* function is designated by dashed line, solid lines correspond to previously described relationships.

Our interpretation is further supported by the recently described localization of kinesin 12 to polycystin 1 (PC1) positive urinary exosome like vesicles [ELVs; [27]] together with *Cys1*, *Pkhd1* and *Pkd2* encoded proteins. In addition, the expression of *Kif12*, *Pkhd1* and *Cys1*, are directly regulated by *HNF1β* [25]. Since *Kif12* expression also strongly correlates with the expression of *HNF1 β*, as well as *Pkhd1* and *Cys1* in the GUDMAP datasets, we propose that these four genes may define a functional complex that together with *Pkd1* [28, 29] modulates the progression of recessive PKD (Fig 6).

Additional supporting evidence implicating *Kif12* as the *Mpkd2* modifier gene candidate is provided by recent studies that demonstrate renal cystic disease-attenuating effects of *Kif3a* deletion in *Pkd1* and *Pkd2* mouse models [30]. Similarities between the N-terminal kinesin motor domain and the C-terminal cargo-binding domain between kinesin 12 and kinesin 3a [31] as well as kinesin 17 [32] suggest that kinesin 12 may complement the function of these motor ciliary proteins. We speculate that the C57BL/6J-associated five amino acid deletion within the kinesin 12 motor domain may impair function of this protein and have cystic disease-inhibiting effects similar to the *Kif3a* deletion. Kinesin 12 may also complement the function of other kinesins that direct biogenesis and function of primary cilia, e.g. through regulation of microtubular dynamics [kinesin 7 [33]] or nucleation of cilia at centrioles [kinesin 24 [34]]. In addition, we note that kinesin 12 is expressed by macrophages [http://www.ncbi.nlm.nih.gov/geo; e.g., GDS3554 and GDS3555 [35], and GDS2430 [36]] and the C57BL/6J-associated deletion may alter their function. Macrophages induce proliferation of cystic epithelial cells in the *Cys1* ^*cpk*^ and *Pkd1* models [37, 38] and their markers are abundantly expressed in kidneys from ARPKD patients [15, 39, 40] as well as *Cys1* ^*cpk/cpk*^ mice carrying the CAST-derived *Kif12* allele [8, 15, 39].

However, it must be noted that unlike the well-studied anterograde IFT kinesins (e.g., kinesin 3a) that are conserved in most eukaryotic cells, kinesin 12 likely emerged later in evolution [41], its orthologues appear only in mammals and birds together with orthologues of *Pkhd1*. *Cys1* so far has only been detected in mammals (based on NCBI Homologene; http://www.ncbi.nlm.nih.gov). Therefore, while the data presented in this report and supporting evidence from other recent studies are intriguing, they provide only circumstantial evidence for *Kif12* as the *Mpkd2* candidate gene. Further rigorous analyses, such targeted gene conversion experiments, are required to validate *Kif12* as the *Mpkd2* gene.

While the impact of modifier genes in modulating the ARPKD phenotype is suggested by intrafamilial variability in disease expression [42–44], this proposition also has yet to be systematically evaluated. Such studies are complicated by compound heterozygosity for the majority of low frequency *PKHD1* mutations. However, a recent study in South African Afrikaners demonstrated a founder effect in which most ARPKD patients are homozygous for a single *PKHD1* mutation and yet the clinical phenotypes in this cohort are variable [45]. Such populations provide powerful experimental resources for future studies to test the impact of *KIF12* and other specific candidate modifier genes on disease progression in human ARPKD. In addition, *KIF12* variants may modulate the severity of a broader spectrum of hepato-renal fibrocystic disorders or even trigger a ciliopathy such as *KIF7* mutation-induced Joubert syndrome [33].

In summary, we have performed functional validation of the renal cystic disease-modulating effects associated with the *Mpkd1-3* interval. We have demonstrated that the CAST-derived *Mpkd1-2* interval promotes cystic disease progression independently of other loci and in a dominantly-acting fashion. We have used *Mpkd1-2* recombinants to fine-map the *Mpkd2* locus to a 14 Mbp interval with 92 RefSeq sequences and developed convergent lines of experimental evidence that implicate *Kif12* as the principal candidate for the *Mpkd2* effects. This work sets the stage for direct hypothesis testing of *Kif12* as a genetic modifier using targeted gene conversion experiments in *cpk* mice, as well as for initial directed studies of *KIF12* as a genetic modifier in specific human ARPKD populations, such as the Afrikaner cohort.

## Materials and Methods

### Mice

The C57BL/6J-*Cys1* ^*cpk/+*^ mice were obtained from the Jackson Laboratory (Bar Harbor, ME). The *Cys1* mutation arose spontaneously on the B6 background. The congenic line carrying the CAST-derived proximal to medial segment of chromosome 4 (B6.CAST.4PM) on a B6 background [17]. This CAST fragment corresponds to the *Mpkd1-3* interval identified by our previous QTL mapping [8]. We have used non-recombinant F2 mice generated from a (B6.CAST.4PM x B6-*Cys1* ^*cpk/+*^)F1 intercross to establish homozygous B6.CAST.4PM-*Cys1* ^*cpk/+*^ and B6.4PM-*Cys1* ^*cpk/+*^ mouse lines. Cystic disease severity in 10-d old *Cys1* ^*cpk/cpk*^ mice from the B6.CAST.4PM-*Cys1* ^*cpk/+*^ cross was compared to those generated in the B6.4PM-*Cys1* ^*cpk/+*^ cross. Scoring of phenotypes and genotyping with *Cys1* ^*cpk*^ allele-specific assay has been previously described [8, 13].

Additional crosses were established to generate the congenic line B6.CAST.4PM.P1, with the proximal Chr 4 QTL interval that contained the *Mpkd1-2* loci. To evaluate the cystic disease-modulating effects of the CAST-derived *Mpkd1-2* interval, we backcrossed *Cys1* ^*cpk/+*^ F1 mice from the B6.CAST.4PM x B6-*Cys1* ^*cpk/+*^ cross to B6-*Cys1* ^*cpk/+*^ mice. The resulting *Cys1* ^*cpk/cpk*^ mice were screened for recombinants containing CAST-derived segments spanning the proximal or distal portion of the *Mpkd1-2* interval.

The integrity of CAST-derived Chr 4 intervals and the remaining B6 genome in these mouse lines was confirmed using a microsatellite marker-based genome scan [8]. Microsatellite makers were also used to fine-map the boundaries of CAST-B6 breakpoints on Chr 4.

All protocols were approved by the University of Alabama at Birmingham Institutional Animal Care and Use Committee. The University of Alabama at Birmingham is fully accredited by the Association for Assessment and Accreditation of Laboratory Animal Care (AAALAC) International.

### Histomorphometry

Paraffin-embedded kidneys were sectioned through the long axis and the hilus and stained with hematoxylin and eosin. The histomorphometry was performed without knowledge of experimental classifications using Bioquant Osteo 2013 Version 13.2.60 image analysis software. The data was collected by examining: (*i*) Total tissue area; (*ii*) Total cyst area (the area and number of cysts in the entire kidney was measured using void and outline filters in the software, the cystic space criteria were set to >4X diameters of the normal proximal tubule spaces; (*iii*) Medullary area (we estimated the boundary by using the glomeruli as cortical controls), (*iv*) Medullary cyst area (by selecting the pixels in the cystic area within the medulla boundary, we defined the medullary cysts using the same void and outline criteria as above; these analyses also provided the medulla cyst count); (*v*) Boundary cysts: to optimize the accuracy of cyst counting, we created an array that allowed us to subtract the cysts from the medulla if over 50% of the area was within the cortex. All other reported data were calculated as derivatives of the above data. All data were independently validated by an additional reader using a different microscope and image analysis software according to a previously described protocol [16].

### Cell culture and Immunostaining

The IMCD-K2 cell line derived from SV40 transformed internal medullary collecting duct cells (mIMCD-K2; [46]) is a well-established renal principal cell line [39]. Cells were plated at confluence and grown in DMEM/F12 medium containing Earle balanced salt solution supplemented with 10% fetal bovine serum, 100 U/ml penicillin, and 100 mg/ml streptomycin (Thermo Fisher Scientific Inc; Waltham, MA), in 5% CO_2_/95% air at 37°C. Immunostaining was performed after 3–4 d in culture.

The mIMCD-K2 cell line was stained using standard laboratory protocols. Briefly, the cells were quickly fixed in -20°C methanol and rehydrated in PBS. We used polyclonal anti-mouse α-tubulin antibody (Invitrogen Corporation, Carlsbad CA) and polyclonal anti-human kinesin 12 (N-18) antibody (Santa Cruz Biotechnology, Santa Cruz, CA). Secondary antibodies were obtained from Invitrogen. Immunostaining was performed after blocking tissue sections for 30 min with PBS containing 1% bovine serum albumin (Sigma). Primary antibody diluted in blocking buffer was incubated with the tissues for 12-hours at 4°C, followed by four rinses with PBS. Nuclei were stained with Hoechst No. 33528 (Sigma) diluted 1:1,000 in PBS, rinsed 3 times in PBS, then mounted in Prolong Gold antifade mounting media (Molecular Probes). A similar approach was applied to immunostaining of a mIMCD cell line stably expressing a GFP-tagged somatostatin receptor 3; *Sstr3*-GFP [26]. Stained samples were analyzed using a fluorescent Leica HC microscope (Leica, Allendale, NJ) and MetaMorph software (Molecular Devices, Sunnyvale, CA).

### GUDMAP analyses

This study utilized data and analytical tools available from the NIDDK GUDMAP developing kidney gene expression atlas database (http://www.gudmap.org; April 2011) including the genome wide gene-expression datasets for developing kidney [22].

### Statistical analyses

Statistical evaluations were performed with SPSS 11.5 statistical software package (SPSS Inc.). Renal cystic phenotypes for both kidneys were combined to give an average phenotypic score for each mouse. Significance of the differences between the two groups was determined by an unpaired, two-tailed t-test.

## Supporting Information

S1 Fig Global genome-wide correlation of gene expression patterns to the one of *Cys1*. Among ~40,000 transcripts profiled in 18 different renal anatomical structures at different developmental time-points that were deposited into the GUDMAP Database, the *Cys1* expression was highly correlated with the *Mpkd2* gene candidate, *Kif12*.
(PNG)
