# Supplementary figures and images for: Genetic and Informatic Analyses Implicate Kif12 as a Candidate Gene within the Mpkd2 Locus That Modulates Renal Cystic Disease Severity in the Cys1cpk Mouse
Source: PLoS One. 2015 Aug 21;10(8):e0135678. doi: 10.1371/journal.pone.0135678 (PMC4546649; doi:10.1371/journal.pone.0135678)

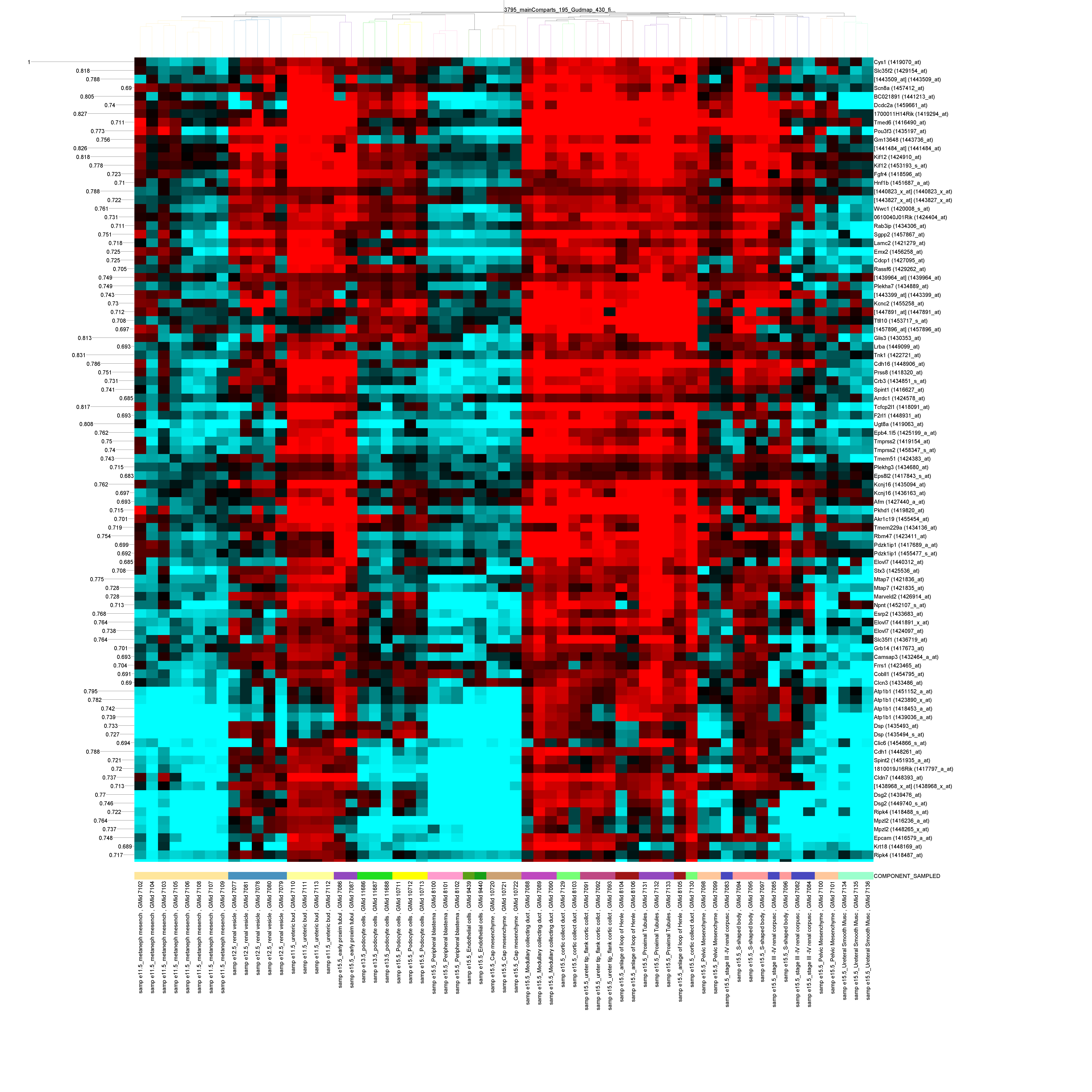

Supplement: S1 Fig — Among ~40,000 transcripts profiled in 18 different renal anatomical structures at different developmental time-points that were deposited into the GUDMAP Database, the Cys1 expression was highly correlated with the Mpkd2 gene candidate, Kif12. (PNG) [file pone.0135678.s001.png]
